# MgZnFe-layered double hydroxides as a dual-function platform for enhancing subunit vaccine efficacy in tumor immunotherapy

**DOI:** 10.1016/j.ijpx.2025.100384

**Published:** 2025-09-01

**Authors:** Xue Wang, Lu Liu, Yingying Chen, Lirui Jia, Yongjun Wang, Wei Jing, Guangqi Yan

**Affiliations:** a School and Hospital of Stomatology, Liaoning Provincial Key laboratory of Oral Diseases, China Medical University, Shenyang 110101, China; b Department of Pulmonary and Critical Care Medicine, Shengjing Hospital of China Medical University, Shenyang 110022, China; c Wuya College of Innovation, Shenyang Pharmaceutical University, Shenyang 110016, China; d Department of Clinical Oncology, Shengjing Hospital of China Medical University, Shenyang 110001, China

**Keywords:** Layered double hydroxide, Endosome lysis, Lymph nodes targeting, Cross-presentation, Cancer vaccine

## Abstract

Therapeutic cancer vaccines show promise in immunotherapy but face challenges such as poor antigen delivery, insufficient immune activation, and an immunosuppressive tumor microenvironment, limiting tumor-specific cellular immunity. In this study, we present a dual-function tumor vaccine platform, MgFeZn-Layered double hydroxide (LMFZ), serving as both an antigen delivery system and immune adjuvant to enhance antitumor responses. Loaded with antigens, nanometer-sized LMFZ particles efficiently traffic to lymph nodes, promoting antigen capture by dendritic cells (DCs). LMFZ endosomal escape enables antigen cross-presentation and enhances DC maturation, boosting cytotoxic T lymphocyte proliferation and tumor infiltration. LMFZ also neutralizes tumor acidity, induces M1 polarization of macrophage, and enhances natural killer cell activity via Zn^2+^ supplementation, amplifying antitumor immunity. Following two peritumoral subcutaneous injections, antigen-loaded LMFZ demonstrated significant antitumor efficacy with minimal adverse effects in a mouse model. This highlights the potential of LMFZ vaccine platform for clinical translation and offers an innovative strategy to address the limitations of current therapeutic vaccine-based immunotherapies.

## Graphical abstract

**Figure:**
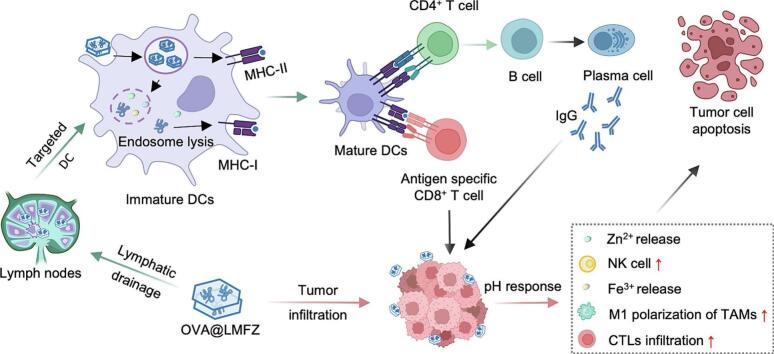

## 1 Introduction

Cancer immunotherapy has become recognized as a transformative strategy, offering renewed hope for effective cancer treatment in recent years (Liu et al., 2022; Laskowski et al., 2022; Ghisoni et al., 2024; Garcia Del Muro et al., 2025). Among its strategies, tumor vaccines stand out for their capacity to introduce tumor-specific antigens, thereby activating T cells within patients' immune systems to precisely identify and eliminate cancer cells (Gonzalez et al., 2018; Singh et al., 2021). This approach is characterized by high specificity, low toxicity, and durable immunological memory, which minimizes off-target effects and supports long-term immune surveillance against cancer recurrence. Moreover, tumor vaccines can be combined with other therapies to further enhance therapeutic outcomes. These advantages collectively position tumor vaccines as a promising strategy with substantial clinical potential in cancer immunotherapy (Lin et al., 2022; Fan et al., 2023). Despite the promise of tumor vaccines, their clinical benefits remain limited. The primary limitations stem from inefficient antigen delivery to lymph nodes and suboptimal immunogenicity of antigen themselves, both of which impair the activation of robust cellular immune responses (Jou et al., 2021; Bowen et al., 2018). As a result, many tumor vaccines often fail to elicit sufficient antitumor activity, resulting in inadequate tumor control and limited long-term remission. Addressing these challenges requires a dual focus: enhancing antigen targeting to lymph nodes and improving antigen immunogenicity (Andorko et al., 2015; Fehres et al., 2014). To this end, optimizing delivery systems to precisely direct antigens to antigen-presenting cells (APCs) within lymph nodes, combined with the development of innovative adjuvants to stimulate potent cytotoxic T lymphocyte (CTL) responses, holds substantial potential for expanding the efficacy of cancer immunotherapy (Zeng et al., 2017; Zhao et al., 2023).

Nanomaterialsare increasingly regarded as hopeful tools in vaccine delivery and immune enhancement, offering substantial advantages in improving antigen delivery efficiency and targeting specificity (Liu et al., 2020). Their unique size, surface properties, and drug-loading capacity allow for efficient trafficking to lymph nodes, thereby enhancing antigen uptake, processing, and presentation (Gong et al., 2024; Wang et al., 2024). Various formulations, including injectable hydrogels (Meng et al., 2021; Yang et al., 2018), polymeric nanoparticles (Xiao et al., 2021; Mei et al., 2018; Wang et al., 2022), exosomes (Zuo et al., 2022), and protein-based nanoparticles (Neek et al., 2019) have been developed. However, few are currently available for clinical use (Xia et al., 2023). Recently, metal-based immunotherapy has achieved remarkable progress as an innovative cancer approach to cancer treatment (Sun et al., 2024). Metal ions and metal-based nanomaterials, known for their immunoactivity and unique physicochemical properties, have garnered considerable attention (Zhong and Sun, 2020; Abánades Lázaro et al., 2024). Among these, layered double hydroxides (LDHs), nanoscale materials composed of hydroxyl complexes of divalent and trivalent metal ions, are strong contenders for vaccine delivery platforms (Abd El-Monaem et al., 2023). Their high surface area and positively charged layered structure create favorable conditions for antigen loading (Abd El-Monaem et al., 2023; Ameena Shirin et al., 2021). Furthermore, LDHs protect antigens from degradation, ensuring effective lymph node targeting and APCs internalization, while also promoting antigen cross-presentation through endosomal escape (Rabiee et al., 2020; Yu et al., 2020). Moreover, the metal ion composition in LDHs can be precisely tailored to endow vaccines with specific adjuvant properties, further modulating and enhancing immune responses. With excellent stablity and biocompatibility, LDHs enable immune activation with minimal side effects (Cunha et al., 2016). Consequently, the development and optimization of LDH-based delivery platform may present valuable opportunities to create safer and more effective tumor vaccines, paving the way for novel strategies in cancer immunotherapy.

As an essential nutrient, zinc (Zn^2+^) serves a pivotal function in the immune system. Zn^2+^ enhances adaptive immunity not only by facilitating the maturation and antigen presentation of DC (Ding et al., 2023), but also directly influences T cell receptor signaling and modulates the Th1/Th2 balance, favoring Th1-mediated cellular immunity (Kaltenberg et al., 2010; Rink and Kirchner, 2000; Chabosseau and Rutter, 2016). Beyond adaptive immunity, Zn^2+^ also contributes to natural killer (NK) cell function by aiding the identification of major histocompatibility complex class I (MHC-I) complexes on target cells (Anfossi et al., 2006), thereby enhancing NK cell differentiation and cytotoxic activity (Muzzioli et al., 2007). Similarly, iron (Fe^3+^) also exerts multifaceted effects on immune regulation, influencing the activity of APCs, T cells and macrophages cells. In APCs, Fe^3+^ enhances intracellular antigen processing and cross-presentation by modulating redox reactions and cellular metabolism (Schaible and Kaufmann, 2004). Balanced iron levels are essential for immune cell functionality, as deficiency inhibits T cell proliferation and differentiation, while adequate levels support energy metabolism and immune activation. Within the tumor microenvironment, Fe^3+^ promotes T cell infiltration and effector functions (Nairz et al., 2010) while supporting M1 macrophage polarization, fostering pro-inflammatory and anti-tumor responses (Jung et al., 2015).

**Fig. 1:**
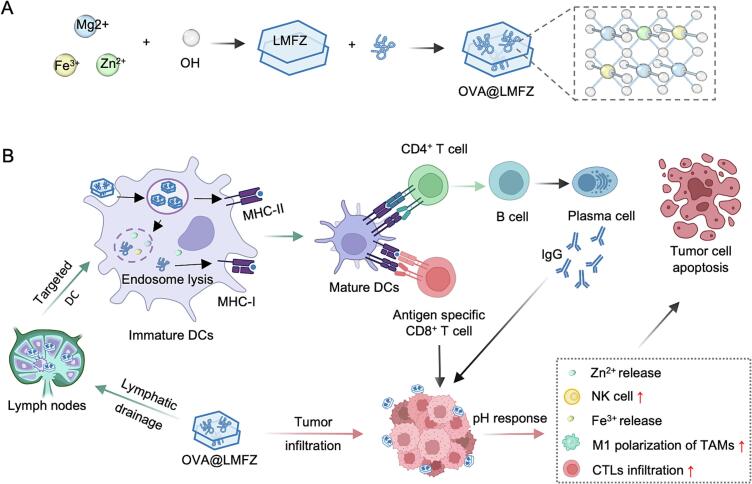
(A) Schematic illustration of the fabrication process of the OVA@LMFZ vaccine. MgFeZn-layered double hydroxide (LMFZ) nanoparticles were synthesized via hydrothermal treatment and subsequently loaded with ovalbumin (OVA) through simple incubation to form OVA@LMFZ. (B) Proposed mechanisms underlying the immunotherapeutic enhancement by OVA@LMFZ. After peritumoral subcutaneous injection, OVA@LMFZ drains to lymph nodes where it is efficiently internalized by dendritic cells (DCs). Inside DCs, the acidic endosomal environment triggers hydrolysis of LMFZ, leading to swelling, rupture, and antigen release into the cytoplasm for cross-presentation via MHC-I, thus activating antigen-specific CD8^+^ cytotoxic T lymphocytes (CTLs). Concurrently, released Zn^2+^ enhances DC maturation and natural killer (NK) cell cytotoxicity, while Fe^3+^ promotes T cell infiltration and macrophage M1 polarization within the tumor microenvironment. Together, these processes induce robust humoral and cellular immune responses and potent antitumor effects.

In this work, we engineered a two-in-one tumor vaccine platform, LMFZ, which serves both as an antigen delivery carrier and immune agonist, leveraging Fe^3+^ and Zn^2+^ to enhance antigen delivery and activate cellular immunity. As illustrated in Fig. 1, LMFZ was prepared through a straighforward hydrothermal method, and ovalbumin (OVA) was loaded onto the layers through a simple incubation process. After peritumoral subcutaneous injection, OVA@LMFZ reached lymph nodes via lymphatic drainage, where it was internalized by DCs located there. Then the hydrolysis of LMFZ within the endosome induced swelling and rupture, releasing the antigen into the cytoplasm to promot cross-presentation and robust cellular immune activation. The activated CTLs subsequently migrate to tumor tissue, where they target and eliminate tumor cells. Additionally, OVA@LMFZ localized around the tumor buffered the acidic tumor microenvironment, creating a supportive environment for cellular immunity and tumor cell destruction. Simultaneously, the release of metal ions from LMFZ stimulated NK cell proliferation and promoted the M1 polarization of tumor-associated macrophages, further enhancing tumor eradication. This two-in-one tumor vaccine platform offers a novel direction for cancer immunotherapy, combining simplicity in preparation with significant enhancement of cellular immune responses. These attributes underscore its considerable potential for clinical applications and its promise as an innovative strategy for advancing cancer immunotherapy.

## 2 Experimental

### 2.1 Materials

The chemicals ZnCl_2_, FeCl_3_·6H_2_O, MgCl_2_·6H_2_O and NaOH were supplied by Tianjin Kemiou Chemical Reagent Co., Ltd. (Tianjin, China). OVA was obtained from Sigma-Aldrich (St. Louis, MO, USA)), while Vadimezan (DMXAA) came from Shanghai Aladdin Biochemical Technology Co., Ltd. (Shanghai, China). FITC-OVA were purchased from Solarbio (Beijing, China), and the SIINFEKL peptide was provided by BIOSS (Beijing, China). Hoechst 33342, and MTT were from Dalian Meilun Biotechnology (Dalian, China). ELISA kits for IgG, IgG1, IgG2a were sourced from Jiang Lai Bio-Technology Co., Ltd. (Shanghai, China). Fluorophore-conjugated anti-mouse antibodies were obtained from BD Biosciences Pharmingen (San Diego, CA, USA). Cell culture reagents were supplied by Gibco (Beijing, China), and the rest of the chemicals were of analytical grade, with deionized water used unless otherwise stated.

### 2.2 Cells and animals

The DC 2.4, B16F10-OVA, and HUVEC cells were sourced from the Cell Bank of the Chinese Academy of Sciences (Shanghai, China). These cells were grown in RPMI-1640 medium, supplemented with 10 % fetal bovine serum (FBS) and 1 % penicillin/streptomycin.

Male C57BL/6 mice, aged 6–8 weeks, were procured from the Animal Experimental Center of Shenyang Pharmaceutical University (Shenyang, China). They were kept under specific pathogen-free (SPF) conditions with free access to water and standard chow. All procedures involving animals were carried out following the protocols authorized by the Ethics Committee of Shenyang Pharmaceutical University (Shenyang, China).

### 2.3 Preparation of LMFZ and drug loading

LMFZ were prepared through a hydrothermal approach. In this process, a solution containing MgCl₂, FeCl₃, and ZnCl₂ at a molar ratio of 5:2:1 was combined with NaOH solution under continuous stirring. The solution pH was gradually increased to approximately 10 by dropwise addition of 0.2 M NaOH while stirring for 30 min at ambient temperature. The suspension was then centrifugated at 10,000 g for 5 min, followed by three washes with deionized water. The washed precipitate was resuspended in water and underwent hydrothermal treatment at 90 °C for 4 h. Finally, the product was vacuum-dried overnight. OVA@LMFZ and FITC-OVA@LMFZ were produced by incubating OVA or FITC-OVA with LMFZ for 30 min at ambient temperature. OD@LMFZ was prepared by incubating DMXAA with LMFZ overnight, followed by a 30-min incubation with OVA. For in vivo applications, all formulations were stabilized by incubating them with a BSA solution at a 2:5 mass ratio.

### 2.4 Characterization of LMFZ

The size distribution and zeta potential of LMFZ were measured using a Nano Zetasizer (Malvern Zetasizer Nano ZS90, UK). The morphology of LMFZ was analyzed with scanning electron microscopy (SEM, GeminiSEM 300, ZEISS, Germany). X-ray diffraction (XRD, SmartLab SE, Rigaku, Japan) was utilized to explore the crystal structure of LMFZ, while energy-dispersive X-ray spectroscopy (EDS) mapping (GeminiSEM 300, ZEISS, Germany) was used to assess the elemental composition and distribution. Inductively coupled plasma optical emission spectrometry (ICP-OES, Agilent 5110, USA) was applied to quantify the zinc content in LMFZ.

The fluorescence intensity of FITC-OVA in the supernatant following centrifugation was quantified using a microplate reader (Thermo Fisher, USA) to assess the encapsulation efficiency of OVA in OVA@LMFZ. The encapsulation efficiency of DMXAA in OD@LMFZ was similarly assessed by determining the content of DMXAA in the supernatant after centrifugation using UV–Vis spectrophotometry.

### 2.5 Cell cytotoxicity and in vitro release

The impact of OVA@LMFZ on the viability of B16F10-OVA and DC2.4 cells was assessed through an MTT assay. Cells (1 × 10^3^/well) were plated in 96-well plates and cultured overnight at 37 °C in 5 % CO₂. After incubation with OVA@LMFZ at concentrations of 0, 20, 50, 100, 200, 500, and 1000 μg/mL for 24 h, 20 μL of MTT solution (5 mg/mL in PBS) was i was added and incubated for 4 h. Afterward, the medium was discarded, and 150 μL of DMSO was added to dissolve the formazan crystals. Absorbance at 570 nm was recorded, and cell viability was calculated relative to the control group.

The release profiles of Zn and OVA from OVA@LMFZ (2 mg/mL), as well as DMXAA from OD@LMFZ (1 mg/mL DMXAA), were studied in vitro using PBS at pH of 7.4 and 5.5. Both formulations were dispersed in PBS and kept at 37 °C with constant agitation at 120 rpm. At predefined time points, aliquots of the suspension were collected and centrifuged at 10,000 *g* for 30 min. The supernatants were carefully retrieved for analysis. Zn concentrations were quantified using ICP-OES/MS, while the release of OVA and DMXAA was quantified with a BCA Protein Assay Kit and UV–visible spectrophotometry, respectively.

### 2.6 Cellular uptake and endosomal escape

DC2.4 and B16F10-OVA cells were plated into 12-well plates at a concentration of 1 × 10^5^ cells per well and incubated overnight at 37 °C under a 5 % CO₂ environment. Subsequently, the cells were exposed to either free FITC-OVA or FITC-OVA@LMFZ (equivalent to 20 μg FITC-OVA) for 1 h at 37 °C. After washing three times with PBS, cells were redispersed in 0.3 mL PBS, and analyzed for cellular uptake by flow cytometry (BD FACSCelesta™, USA).

To evaluate the cytosolic delivery capability of LMFZ, DC2.4 and B16F10-OVA cells were plated in confocal dishes and exposed to FITC-OVA@LMFZ (equivalent to 20 μg FITC-OVA) for 15, 30, or 60 min at 37 °C. After incubation, cells were marked with Hoechst 33342 to highlight the nuclei and LysoTracker Red to stain endosome/lysosome, and the intracellular distribution of FITC-OVA was observed using a confocal laser scanning microscope (Zeiss LSM 510 Meta, Germany).

### 2.7 In vitro activation of DCs

DC2.4 cells were plated in 12-well plates and treated with either free OVA or OVA@LMFZ (20 μg OVA or 100 μg LMFZ containing 20 μg OVA) for 24 h. After treatment, the cells were rinsed with PBS and stained with PE-labeled anti-CD80 and BV421-labeled anti-CD86 antibodies. Flow cytometry was used to analyze CD80 and CD86 expression, indicating the activation of DC2.4 cells.

### 2.8 In vitro antigen cross-presentation assay

DC2.4 cells were plated in 12-well plates using the same procedure as described in the section on Cellular Uptake. The cells were treated with free OVA or OVA@LMFZ (20 μg OVA or 100 μg LMFZ containing 20 μg OVA) for a duration of 16 h. Following incubation, the cells were rinsed three times with PBS and marked with a PE-conjugated anti-H-2Kb-SIINFEKL antibody to identify SIINFEKL peptide presentation on MHC-I molecules. Flow cytometry was used to evaluate antigen cross-presentation.

### 2.9 Lymphatic targeting and DC activation of OVA@LMFZ in vivo

To assess the lymphatic targeting effectiveness of OVA@LMFZ, a subcutaneous injection of OVA@LMFZ containing 20 μg FITC-OVA was given to the mice. The mice were euthanized twelve hours after injection, and their inguinal lymph nodes were harvested, sectioned, stained with DAPI, and examined using confocal laser scanning microscopy.

To evaluate the uptake of OVA@LMFZ by DCs in lymph nodes, mice subcutaneously injected with either free FITC-OVA or FITC-OVA@LMFZ (equivalent to 20 μg FITC-OVA). Twelve hours later, the inguinal lymph nodes were excised after the mice were euthanized. Single-cell suspensions were generated by filtering the lymph nodes through a 70-μm sieve. The cells were then marked with APC-anti-mouse CD11c and PerCP-Cy5.5-anti-mouse CD8a antibodies and assessed by flow cytometry.

To evaluate the in vivo stimulation of DCs by OVA@LMFZ, mice were given subcutaneous injections of either free OVA or OVA@LMFZ (20 μg OVA or 1 mg MLDHs loaded with 20 μg OVA). Inguinal lymph nodes were obtained three days after the injection, converted into single-cell suspensions, and marked with APC-labeled anti-CD11c, PE-labeled anti-CD80 and BV421-labeled anti-CD86 antibodies and analyzed using flow cytometry.

### 2.10 Tumor penetration of OVA@LMFZ

Mice were subcutaneously injected with 5 × 10^5^ B16F10-OVA cells in the right posterior flank. Once the tumors grew to a volume of around 200 mm^3^, the mice received subcutaneous injections of 100 μL FITC-OVA@LMFZ, containing 20 μg FITC-OVA. Twenty-four hours later, tumors were harvested, cryosectioned, stained with DAPI, and examined for fluorescence distribution using a confocal laser scanning microscope. The Zn^2+^ content in the tumor was also measured using ICP-OES.

### 2.11 LMFZ buffering of tumor acidic pH

Subcutaneous injections of 5 × 10^5^ B16F10-OVA cells were administered to mice in the right posterior flank. Once the tumor volumes reached 200 mm^3^, the mice were injected with either free OVA or OVA@LMFZ (20 μg OVA or 1 mg LMFZ loaded with 20 μg OVA) around the tumor. Twenty-four hours later, SNARF-1 (250 μg/kg), a pH-sensitive fluorescent dye, was administered intravenously. Thirty minutes post-injection, tumors were harvested, cut in half, and analyzed for fluorescence signals associated with pH changes using an In Vivo Imaging System (FX Pro).

### 2.12 In vivo immune response of OVA@LMFZ

Subcutaneous injections of 5 × 10^5^ B16F10-OVA cells were administered to mice in the right posterior flank. Tumor volumes were monitored and, when they reached approximately 80 mm^3^, 100 μL of either free OVA or OVA@LMFZ (20 μg OVA) was administered peritumorally on Day 0, with booster doses given on Day 4.

To assess the OVA-specific antibody response, blood samples were drawn on Day 11 from the angular vein. After centrifugation to separate the serum, the titers of OVA-specific antibodies were determined using an ELISA kit.

T cell activation in the spleen was evaluated on Day 11. Mice were sacrificed, and spleens were harvested and dissociated into single-cell suspensions. Red blood cells were lysed using ACK buffer, and splenocytes were stimulated with OVA (100 μg/mL) or SIINFEKL (2 μg/mL) at 37 °C for 6 h. The cells were then stained with FITC-CD3 and APC-CD4 antibodies or FITC-CD3 and PerCP-Cy5.5-CD8a antibodies, followed by analysis using flow cytometry.

To assess immune cell activation within tumor tissues, mice were euthanized on Day 8. Tumor tissues were chopped and incubated in RPMI 1640 with 0.5 mg/mL collagenase, 0.1 mg/mL hyaluronidase, and 0.1 mg/mL DNase at 37 °C for 1 h to generate single-cell suspensions. T cell populations were identified through staining with FITC-CD3, APC-CD4, and PerCP-Cy5.5-CD8 antibodies. Natural killer (NK) cell levels were analyzed using AF700-NK1.1 and PE-CD107a antibodies. Tumor-associated macrophages (TAMs) were classified as M1 macrophages (labled with FITC-CD11b, BV421-F4/80, and PE-CD80) or M2 macrophages (labled with FITC-CD11b, BV421-F4/80, and APC-CD206).

### 2.13 Antitumor efficacy and safety in vivo

To evaluate the therapeutic effectiveness of OVA@LMFZ and its combined effect with vascular disruption therapy, a bilateral tumor model was developed using male C57BL/6 mice. Mice were injected subcutaneously with 5 × 10^5^ B16F10-OVA cells suspended in 100 μL PBS into the right posterior flank to establish the primary tumor. The next day, the same number of B16F10-OVA cells was injected into the left posterior flank to develop distant tumors. Once the primary tumor volume reached 80 mm^3^, mice were randomly assigned to receive one of the following treatments on Days 5 and 9: PBS, OVA, DMXAA, OVA@LMFZ, or OD@LMFZ (containing 20 μg OVA, 1 mg LMFZ, and 1 mg DMXAA). Tumor size (length and width) and body weight were recorded every two days. Tumor volume was calculated using the formula: Tumor volume (mm^3^) = 1/2 × length × width^2^. When the tumor length exceeded 20 mm or the tumor volume reached 2000 mm^3^, euthanasia was performed on the mice. The endpoint date for each mouse was documented, and Kaplan-Meier survival analysis was conducted to assess the survival probability for each group.

To investigated the local and systemic toxicity of OVA@LMFZ and OD@LMFZ, mice were administered the respective vaccine formulations on Days 5 and 9. Blood samples were collected on Day 13 from the angular vein and centrifuged at 2000 *g* for 10 min at 4 °C to obtain serum. Serum levels of aspartate aminotransferase (AST), alanine aminotransferase (ALT), blood urea nitrogen (BUN), and creatinine (CREA) were analyzed through standard biochemical assays to evaluate hepatotoxicity and nephrotoxicity. Skin samples from the injection sites were harvested, cryosectioned, and underwent staining with hematoxylin and eosin to assess local toxicity. Histopathological analysis was conducted to examine any tissue damage or inflammation resulting from the vaccine formulations.

To assess the distribution and accumulation of LMFZ in normal tissues, mice were given a subcutaneous dose of FITC-OVA@LMFZ, which contained 20 μg FITC-OVA, on Days 5 and 9. On Day 13, the heart, liver, spleen, lungs, and kidneys were collected and analyzed using the IVIS Spectrum imaging system (PerkinElmer, Waltham, MA). Fluorescence was detected and quantified to determine the presence and distribution of the formulations in major organs.

### 2.14 Statistics

Statistical analysis was performed using GraphPad Prism 9 (GraphPad Software, San Diego, CA, USA). Data are presented as mean ± SD. A two-tailed Student's *t*-test compared two groups, while one-way ANOVA was used for multiple groups. Statistical significance was set at *p* < 0.05. All experiments were done in triplicate or more, with sample sizes provided in the figure legends or main text.

## 3 Results

### 3.1 Preparation and characterization of LMFZ and OVA@LMFZ

**Fig. 2:**
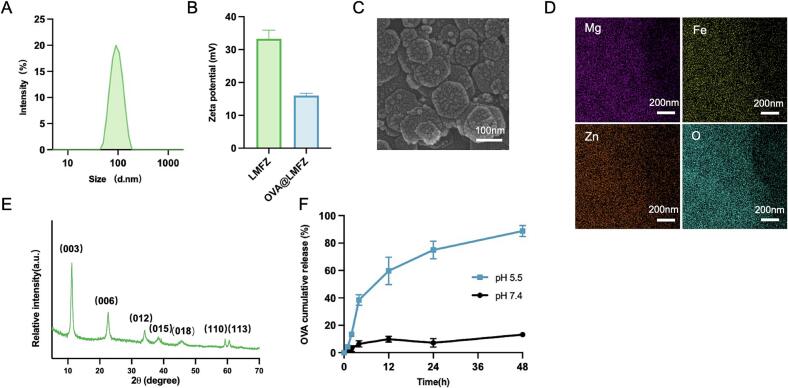
Characterization of LMFZ and OVA@LMFZ. (A) Particle size distribution of LMFZ nanoparticles measured by dynamic light scattering (DLS). (B) Zeta potential of LMFZ and OVA@LMFZ measured by laser Doppler velocimetry. (C) Scanning electron microscopy (SEM) image showing the hexagonal layered morphology of LMFZ. Scale bar: 100 nm. (D) Energy-dispersive X-ray spectroscopy (EDS) mapping revealing uniform distribution of Mg, Fe, and Zn elements within LMFZ. Scale bar: 200 nm. (E) X-ray diffraction (XRD) pattern of LMFZ indicating characteristic LDH crystalline peaks. (F) Cumulative release profile of OVA from OVA@LMFZ at pH 5.5 and pH 7.4 in PBS at 37 °C over 48 h (*n* = 3). Data are presented as mean ± SD.

LDH nanoparticles composed of hydroxy complexes of Mg^2+^, Fe^3+^and Zn^2+^ (LMFZ) were successfully prepared via a hydrothermal method. The hydrodynamic diameters of LMFZ determined, as measured by dynamic light scattering (DLS), was 94.80 ± 1.678 nm (Fig. 2A and Table S1). Moreover, the LMFZ demonstrated a positive zeta potential of +33 mV (Fig. 2B), indicating good colloidal stability (Fig. S1A). The image of scanning electron microscopy exhibited a hexagonal layered structure of LMFZ (Fig. 2C). EDS mapping revealed the uniform distribution of Mg, Fe and Zn ions throughout the LMFZ (Fig. 2D). XRD analysis of LMFZ revealed the presence of characteristic peaks consistent with the crystalline LDH framework (Fig. 2E). The calculated interlayer distance (d-spacing) derived from the (003) peak was around 7.86 Å, which supports the presence of intercalated carbonate ions within the structure. The absence of any supplementary peaks indicates the phase purity and high crystallinity of LMFZ, properties that are advantageous for antigen loading. Moreover, ICP-OES analysis demonstrated that the Zn^2+^ content per gram of LMFZ was 73.92 μg. Collectively, these results indicate that the LMFZ nanoparticles possess favorable physicochemical properties, including optimal size and structural integrity, making them well-suited for antigen delivery and vaccine applications.

The loading of OVA into LMFZ occurred via simple incubation, leading to the formation of OVA@LMFZ. DLS and laser Doppler velocimetry analyses indicated that the encapsulation process led to an increase in the particle size of LMFZ to 121.3 nm (Table S1), along with a 15 mV decrease in zeta potential, while still maintaining a positive surface charge (Fig. 2B). At an OVA-to-LMFZ mass ratio of 1:5, OVA@LMFZ demonstrated an encapsulation efficiency exceeding 80 %. The release kinetics of OVA and Zn^2+^ from OVA@LMFZ were evaluated in PBS at pH values of 5.5 and 7.4 (Fig. 2F, S1B and S1C). At physiological pH 7.4, OVA@LMFZ exhibited minimal release, with only 12 % of OVA and approximately 2 % of Zn^2+^ released over 48 h. This indicates that LMFZ retains a strong association with OVA at neutral pH, effectively preventing off-target antigen release. In contrast, at a more acidic pH (5.5), the release rate markedly increased, with the cumulative release of Zn^2+^ and OVA at 48 h enhanced 2- and 6-fold, respectively. These results highlight the pH-sensitive characteristics of OVA@LMFZ, enabling controlled antigen and ion release in acidic conditions such as those found in endosomes or tumor microenvironments.

### 3.2 OVA@LMFZ facilitates antigen uptake and buffers pH decrease in vitro

**Fig. 3:**
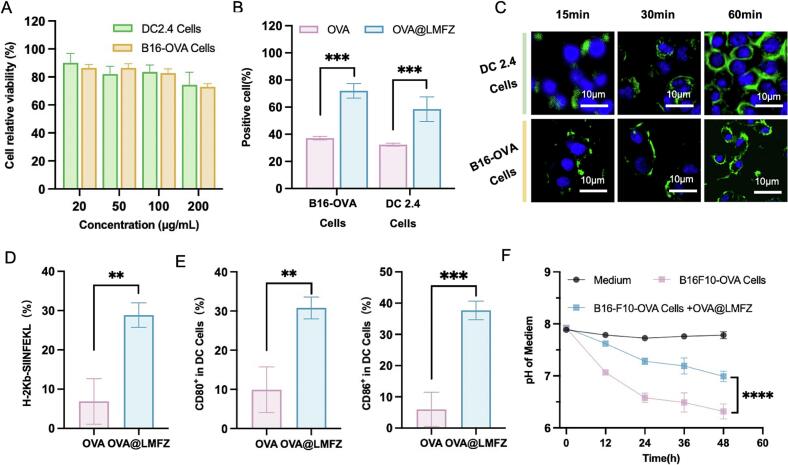
In vitro evaluation of OVA@LMFZ uptake, endosomal escape, cross-presentation, and pH buffering. (A) Cell viability of DC2.4 and B16F10-OVA cells treated with increasing concentrations of OVA@LMFZ (0–100 μg/mL) for 24 h, assessed by MTT assay (*n* = 3). (B) Flow cytometry analysis of cellular uptake efficiency of FITC-OVA and FITC-OVA@LMFZ (equivalent to 20 μg OVA) by DC2.4 and B16F10-OVA cells after 1 h incubation. (C) Confocal laser scanning microscopy images of DC2.4 cells treated with FITC-OVA@LMFZ (20 μg OVA equivalent) for 15, 30, and 60 min. Green: FITC-OVA; blue: nuclei stained with Hoechst 33342. Scale bar: 10 μm. (D) Antigen cross-presentation in DC2.4 cells evaluated by flow cytometry using PE-conjugated anti-H-2Kb-SIINFEKL antibody after 16 h incubation with free OVA or OVA@LMFZ (20 μg OVA) (n = 3). (E) Expression of co-stimulatory molecules CD80 and CD86 on DC2.4 cells measured by flow cytometry after 24 h treatment with free OVA or OVA@LMFZ (20 μg OVA) (n = 3). (F) Change in pH of B16F10-OVA cell culture medium after 48 h incubation with or without OVA@LMFZ (100 μg/mL) (n = 3). *P < 0.05, **P < 0.01, ***P < 0.001. (For interpretation of the references to colour in this figure legend, the reader is referred to the web version of this article.)

The cytotoxicity of OVA@LMFZ was first evaluated in B16F10-OVA and DC2.4 cells using the MTT assay. OVA@LMFZ exhibited low cytotoxicity in both cell types, even at concentrations as high as 100 μg/mL, with cell viability remaining above 80 % (Fig. 3A). In light of these findings, a concentration of 100 μg/mL was selected for further cellular investigations. Then we evaluated the antigen uptake and cross-presentation efficiency of OVA@LMFZ, as well as its ability to buffer pH decreases in tumor cell culture medium. To assess internalization efficiency, DC2.4 cells were treated with either free FITC-OVA or FITC-OVA@LMFZ, and analyzed by flow cytometry. The results revealed that cells treated with OVA@LMFZ exhibited greater uptake than those treated with free OVA (Fig. 3B), indicating that loading OVA onto LMFZ enhances antigen uptake. Confocal microscopy was used to analyze the intracellular trafficking of nanoparticles in DC2.4 cells after internalization. As shown in Fig. 3C, punctate green fluorescence was seen in the cells 15 min after incubation with FITC-OVA@LMFZ, indicating its uptake into endosomes. At 30 min, the fluorescence began to disperse, and by 60 min, it was diffusely distributed throughout the cytoplasm. This distribution pattern suggests that the proton sponge effect caused by LMFZ led to endosomal swelling and eventual rupture, releasing FITC-OVA into the cytosol and allowing the fluorescence to spread.

Exogenous antigens uptaked by DCs are typically processed within phagosomes, where they are associated with MHC-II and presentated to CD4^+^ T cells. In contrast, CD8^+^ T cells recognize antigens peptides presented by MHC-I. For exogenous antigens to activate CD8^+^ T cells response, they must escape the phagosome, enter the cytoplasm, and undergo cross-presentation via the MHC-I pathway. To determine whether LMFZ enhances this process, we examined the surface expression of H-2Kb-SIINFEKL complexes on DC2.4 cells. The datas revealed that the protortion of H-2Kb-SIINFEKL-positive cells was 3.2 times higher following treatment with OVA@LMFZ compared to free OVA (Fig. 3C). These findings demonstrate that LMFZ facilitate antigen uptake, promote endosomal escape, and significantly increase the efficiency of antigen cross-presentation. Moreover, LMFZ markedly increased the expression of costimulatory molecules CD80 and CD86 on DCs in comparison with free OVA (Fig. 3E), highlighting the potential of LMFZ as a potent adjuvant for enhancing dendritic cell activation and subsequent immune responses.

Similar increase in uptake and fluorescence changes in confocal microscopy images were also observed in B16F10-OVA cells after incubation with OVA@LMFZ (Fig. 3B), indicating that OVA@LMFZ can be efficiently taken up by tumor cells, undergo endosomal rupture, and disperses into the cytoplasm for metal ions release and immunological enhancement instead of lysosomal degradation (Fig. 3C and Fig. S6). Furthermore, OVA@LMFZ demonstrated the ability to mitigate the acidification of the culture medium. As shown in Fig. 3F, following 48 h of incubation in blank medium, B16F10-OVA cells decreased the pH of the medium by approximately 1.6. In contrast, with the addition of OVA@LMFZ to the medium, the pH dropped by only 0.9 during the same period. This significant buffering effect alleviates the acidification caused by tumor cell metabolism, potentially improving the immunosuppressive tumor microenvironment and creating conditions more favorable for immune activation.

### 3.3 OVA@LMFZ targets lymph nodes and activates DCs in vivo

**Fig. 4:**
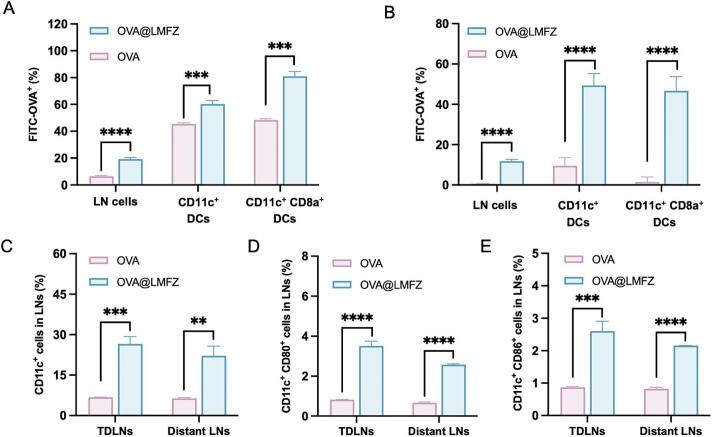
In vivo lymph node targeting and dendritic cell activation by OVA@LMFZ. (A-B) Percentages of FITC-OVA positive cells, CD11c^+^ DCs, and CD11c^+^ CD8a^+^ DCs in tumor-draining lymph nodes (TDLNs) and distant lymph nodes (LNs) 12 h post subcutaneous injection of free FITC-OVA or FITC-OVA@LMFZ (20 μg OVA equivalent) (*n* = 3). (C) Percentage of CD11c^+^ DCs, (D) CD11c^+^ CD80^+^ DCs, and (E) CD11c^+^ CD86^+^ DCs in TDLNs and distant LNs measured by flow cytometry 72 h post injection of free OVA or OVA@LMFZ (20 μg OVA) (n = 3). Data are presented as mean ± SD. **P < 0.01, and ***P < 0.001.

For a robust effector T cell response, vaccines must be captured by APCs located in or migrating to lymph nodes to trigger adaptive immunity. To analyze the lymph node-targeting capacity of OVA@LMFZ, the nanoparticles were administered subcutaneously around the tumor in mice. After FITC-OVA@LMFZ treatment for 12 h, tumor-draining lymph node (TDLNs) sections were dyed with DAPI and observed using fluorescence microscopy. The images revealed the colocalization of green fluorescence from FITC-OVA with lymph node cells, indicating the effective migration of FITC-OVA@LMFZ from the injection site to the lymph nodes (Fig. S2). Flow cytometry was used to examine FITC-OVA-positive cells in lymph nodes and to assess the uptake efficiency of FITC-OVA@LMFZ by lymph node-resident cells, particularly DCs. Whether in TDLNs or distant lymph nodes, a much higher proportion of FITC-OVA-positive cells was observed in the FITC-OVA@LMFZ-treated group in comparison with free OVA-treated group (Fig. 4A and B). As expected, the percentages of CD11c^+^ DCs in FITC-OVA positive cells were raised to as high as 60 % and 49 % by LMFZ in TDLNs and distant lymph nodes, respectively (Fig. 4A and B). Furthermore, the proportion of CD8a^+^ subpopulations responsible for antigen cross-presentations in CD11c^+^ DCs that have internalized FITC-OVA were significantly elevated after OVA@LMFZ treatment in both types of lymph nodes. These results indicate that OVA@LMFZ efficiently delivers antigens to lymph node-resident APCs, particularly CD11c^+^ CD8a^+^ DCs, facilitating efficient antigen capture and cross-presentation in vivo.

For DCs to effectively activate T cell responses, they must not only present antigens but also upregulate the expression of costimulatory molecules. The ability of OVA@LMFZ to promote DC activation and expansion within lymph nodes was therefore evaluated. Compared to free OVA group, the percentage of CD11^+^ DCs in TDLNs and distal lymph nodes of mice treated with OVA@LMFZ increased by 2.9-fold and 2.5-fold, respectively (Fig. 4C), indicating that LMFZ promoted DC proliferation. Furthermore, in the OVA@LMFZ-treated group, the proportions of CD11c^+^CD80^+^ DCs and CD11c^+^CD86^+^ DCs in lymph nodes (Fig. 4D) were considerably higher than those observed in the free OVA group. This indicates that LMFZ promotes extensive DC activation and proliferation, further supporting its role in facilitating the stimulation of robust cellular immune responses. The above results collectively indicate that OVA@LMFZ effectively targets lymph nodes and promotes DC activation, underscoring the potential of OVA@LMFZ as a potent vaccine adjuvant for enhancing immune responses.

### 3.4 OVA@LMFZ activates immune responses

**Fig. 5:**
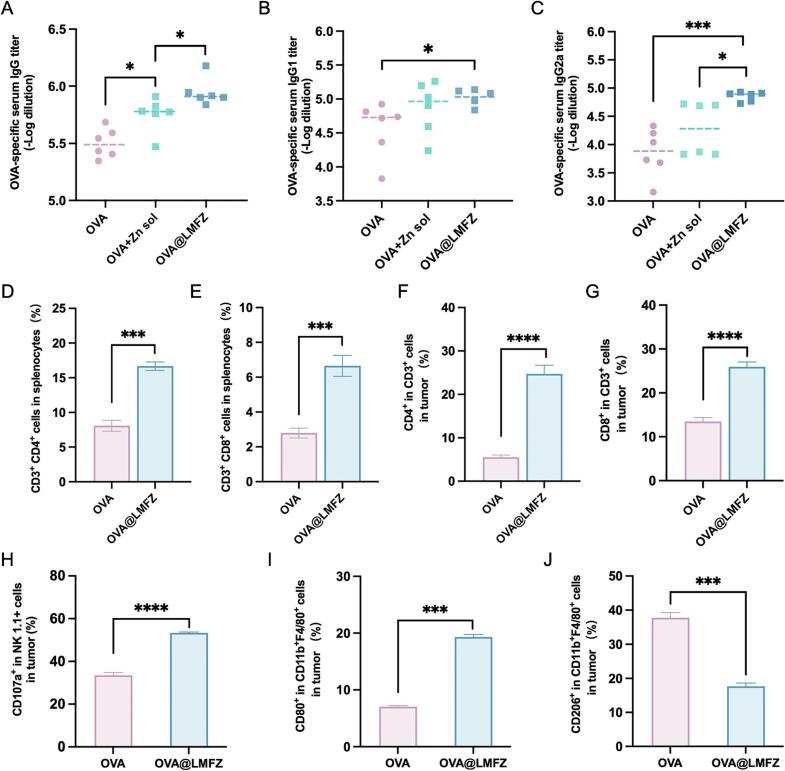
Humoral and cellular immune responses activated by OVA@LMFZ. (A-C) Serum titers of OVA-specific IgG, IgG1, and IgG2a in vaccinated mice measured by ELISA on Day 11 post immunization (*n* = 3). (D) Percentage of OVA-specific CD4^+^ T cells in splenocytes after restimulation with OVA (100 μg/mL) and (E) CD8^+^ T cells after SIINFEKL (2 μg/mL) stimulation, analyzed by flow cytometry on Day 11 (n = 3). (F-G) Tumor-infiltrating CD4^+^ and CD8^+^ T cell subsets, (H) activated NK cells (CD107a^+^ NK1.1^+^), (I) M1-like tumor-associated macrophages (CD11b^+^ F4/80^+^ CD80^+^), and (J) M2-like TAMs (CD11b^+^ F4/80^+^ CD206^+^) in tumors harvested on Day 8 post treatment, assessed by flow cytometry (n = 3). Data are presented as mean ± SD. *P < 0.05, **P < 0.01, ***P < 0.001.

We assessed the in vivo antigen-specific immune responses triggered by OVA@LMFZ. The results demonstrated that OVA@LMFZ induced markedly enhanced antibody responses compared to those appeared in mice treated with free OVA (Fig. 5A, B and C). OVA@LMFZ prominently induced higher titers of IgG and IgG2a compared to the OVA + Zn sol group (Fig. 5A and C), suggesting that embedding OVA within LMFZ substantially boosts its immunogenicity, particularly by enhancing cellular immunity.

OVA@LMFZ induced more potent T cell activation, as expected, showing a substantial rise in both CD4^+^ T cells and CD8^+^ T cells within the spleen compared to the free OVA group (Fig. 5D and E). These findings indicate that OVA@LMFZ greatly enhances antigen-specific cellular and humoral immune responses in vivo.

Beyond targeting the lymph nodes, the injected OVA@LMFZ also permeated into the tumor tissue (Fig. S3A and S3C). The hydrolysis of OVA@LMFZ consumed excess H^+^ within the tumor microenvironment, thereby preventing excessive acidification (Fig. S3B) and simultaneously releasing metal ions. An increased proportion of anti-tumor M1 macrophages and a decreased population of pro-tumor M2 macrophages were observed in the OVA@LMFZ immunization group (Fig. 5I and J). The findings imply that neutralization of the tumor microenvironment, coupled with metal ion supplementation, promotes macrophages polarization toward the pro-tumor M1 phenotype. Additionally, these effects supported enhanced infiltration of T cells and activation of NK cells, both of which are essential for tumor eradication. The proportions of tumor-infiltrating CD4^+^ and CD8^+^ T cell subsets increased by 3.5-fold and 0.9-fold, respectively (Fig. 5F and G), consistent with the activation and proliferation observed in the spleen (Fig. S3C and 5H). Furthermore, the release of Zn^2+^ by OVA@LMFZ mya enhance cGAS sensitivity, activating the STING pathway, as evidenced by elevated serum levels of IFN-β (Fig. S3D).

### 3.5 OVA@LMFZ triggers potent antitumor immunotherapy

**Fig. 6:**
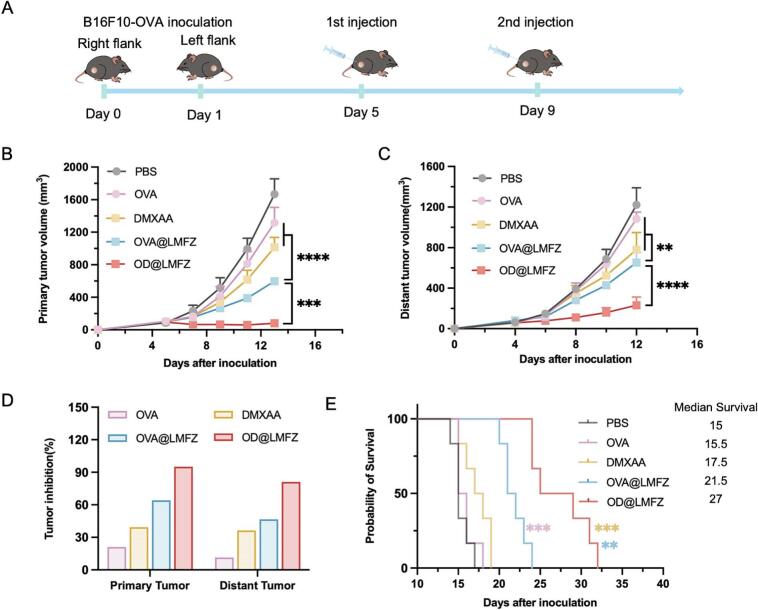
Antitumor efficacy and survival benefits of OVA@LMFZ and OD@LMFZ in a bilateral B16F10-OVA tumor model. (A) Experimental scheme: C57BL/6 mice were implanted with B16F10-OVA cells on Days 0 (right flank) and 1 (left flank), and treated with indicated formulations on Days 5 and 9. (B) Primary tumor growth curves, (C) distant tumor growth curves, (D) percentage tumor inhibition rates, and (E) Kaplan-Meier survival curves for mice treated with PBS, free OVA, DMXAA, OVA@LMFZ, or OD@LMFZ (*n* = 6). *P < 0.05, **P < 0.01, and ***P < 0.001; Purple** versus OVA+Zn sol; Yellow * versus DMXAA and Blue* versus OVA@LMFZ. (For interpretation of the references to colour in this figure legend, the reader is referred to the web version of this article.)

Given the enhancing effects of OVA@LMFZ on humoral and cellular immunity, we investigated its efficacy in inhibiting tumor growth using a bilateral B16F10-OVA murine model. Briefly, C57BL/6 mice received B16F10-OVA cell implants on their right and left posterior flanks on days 0 and 1, respectively, and were immunized on days 5 and 9 (Fig. 6A). The data revealed that OVA@LMFZ treatment caused a marked decrease in the growth of both primary and distant tumors compared to free OVA (Fig. 6B and C). Tumor inhibition rates in the OVA@LMFZ group reached 46.5 % for primary tumors and 64.1 % for distant tumors (Fig. 6D), likely driven by the enhanced cellular immune responses elicited by the platform. Correspondingly, the median survival time in the OVA@LMFZ treatment group was extended to 21.5 days, a substantial improvement compared to the 15.5 days observed in the OVA treatment group (Fig. 6E).

The suppressive effect of OVA@LMFZ on tumor progression and the extension of overall survival in tumor-bearing mice can be further enhanced by loading the vascular disrupting agent DMXAA. The OD@LMFZ formulation was created by loading DMXAA onto LMFZ through ion exchange. A red shift of the maximum ultraviolet absorption wavelength of DMXAA confirms its successful loading onto LMFZ (Fig. S4A). The loading capacity of OD@LMFZ was 50 % and the encapsulation efficiency reached 70.95 %. Under weakly acidic conditions (pH 5.5), DMXAA can be effectively released from LMFZ, with a cumulative release amount of approximately 60 % over 48 h. The release rate is also significantly higher under acidic conditions than at pH 7.4, indicating that LMFZ provides stable protection for DMXAA under physiological conditions while enabling effective release in the weakly acidic environment, like endosomes, to achieve therapeutic effects (Fig. S4B). Following two administrations, OD@LMFZ treatment achieved tumor inhibition rates of 95 % and 81 % for primary and distant B16F10-OVA tumors in mice, respectively (Fig. 6B, C and D). Additionally, the median survival time was extended to 27 days, with significant therapeutic benefit over both DMXAA and OVA@LMFZ treatment group (Fig. 6E). These results emphasize the therapeutic promise of OD@LMFZ in boosting its inhibitory effect on tumor and increasing survival duration. The H&E staining imaging of the in situ tumor tissue sections of mice at the end of the treatment was consistent with the above efficacy results (Fig. S7).

### 3.6 Safety evaluation of OVA@LMFZ and OD@LMFZ

The systemic and local safety of OVA@LMFZ and OD@LMFZ formulations was thoroughly assessed to confirm their tolerability. Both treatments demonstrated excellent safety profiles, with no observable adverse effects. Body weights of B16F10-OVA tumor-bearing C57BL/6 mice remained stable throughout the study, and serum biochemical markers (ALT, AST, BUN, and CREA) indicated normal liver and kidney function (Fig. S5A and S5B). Compared to the PBS control, histological analysis of skin samples from injection sites showed intact tissue structures without any noticeable damage in all groups (Fig. S5C). The evaluation of tissue distribution using IVIS Spectrum imaging showed no significant fluorescence in major organs, including the heart, liver, spleen, lungs, and kidneys, following subcutaneous injection of FITC-OVA@LMFZ (Fig. S5D). The findings indicate that the vaccination protocol and dosing regimen of OVA@LMFZ and OD@LMFZ are well-tolerated, causing neither local nor systemic toxicity.

## 4 Discussion

Leveraging nanotechnology to targete antigens and adjuvants to lymph nodes has become a highly effective strategy for initiating and enhancing immune responses (De Koker et al., 2016; Kaminskas and Porter, 2011). In this study, we developed a dual-function tumor platform, LMFZ, designed to effectively deliver both antigen and adjuvant to lymph nodes. LMFZ was synthesized by simply mixing a NaOH solution with a metal ion salt solution, followed by hydrothermal treatment. This resulting nanomaterial is composed of stacked hexagonal lamellar structure formed by alternating hydroxy complexes of Mg, Zn, and Fe, as shown in Fig. 2C and D. The material exhibits a high specific surface area, which is advantageous for antigen loading. Unlike many previously described systems, such as gold nanoparticles (Peipei Zhang et al., 2015; Tao et al., 2015), micellar nanoparticles (Wilson et al., 2013), and lipid‑calcium phosphate nanoparticles (Xu et al., 2013), which often require chemical conjugation of the antigen to the carriers, our LMFZ platform simplifies the production process. Chemical conjugation can complicate the production process and may reduce the antigen's activity, whereas LMFZ achieves high antigen loading efficiency through a simple incubation process. The loading mechanism is as follows: Firstly, the negatively charged carboxyl residues on the OVA surface electrostatically interact with the positively charged lamellar structure of LMFZ, enabling OVA to bind to the LMFZ surface. Secondly, negatively charged OVA molecules enter the interlayer channels of LMFZ through an ion-exchange mechanism. Additionally, hydrogen bonding between the carboxyl residues of OVA and the hydroxyl groups of the LMFZ lamellar structure, along with hydrophobic interactions, further enhance the antigen loading capacity. When the mass ratio of OVA to LMFZ is 1:5, the encapsulation efficiency exceeds 80 %.

Smaller nanocarriers tend to exhibit greater efficiency in reaching lymph nodes compared to their larger counterparts (Reddy et al., 2006). Nanoparticles ranging from 20 to 200 nm can directly access lymphoid tissues through the lymphatic drainage system within a few hours after injection. In contrast, larger particles (200–500 nm) rely on DCs for migration to lymph nodes, a process that typically takes around 24 h (Kedmi et al., 2010; Manolova et al., 2008). The particle size of LMFZ was approximately 95 nm, and after antigen loading, it only increased slightly to 121 nm (Table S1). This minimal size increase allowed OVA@LMFZ to maintain efficient migration to lymph nodes, as demonstrated by the fluorescence of FITC-OVA observed in lymph nodes 12 h after administration in mice (Fig. S2). These findings suggested that the LMFZ not only protect OVA from degradation but also facilitates its efficient delivery to secondary lymphoid organs, enabling persistent antigen stimulation for robust immune activation.

Upon reaching the lymph nodes, antigens must be internalized by DCs, processed, and presented via MHC-I molecules on the cell surface to trigger CD8^+^ T cell-mediated cellar immune response. This presents a challenge in designing nanoparticle-based vaccines, as MHC-I cross-presentation of exogenous antigens requires the release of antigens into the cytoplasm, bypassing the lysosomal degradation pathway. In our study, OVA@LMFZ facilitated the efficient uptake of OVA by lymph node-resident DCs, particularly CD8a^+^ DCs, which play a key role in cross-presentation (Fig. 4A and B). The superior performance of OVA@LMFZ compared to free OVA may be attributed to the positive charge on OVA@LMFZ (Fig. 2B), which serves as a “danger signal” to stimulate DCs. This observation aligns with previous findings where antigens encapsulated in positively charged DOPC liposomes exhibited enhanced antigen-specific immune response (Kim et al., 2015). After internalization by DCs, LMFZ interacts with H^+^ ions in the acidic endosome, undergoing hydrolysis and releasing substantial amounts of Zn^2+^ and Fe^3+^. This process triggers an influx of Cl^−^ ions and water molecules, leading to endosomal disruption (Xu et al., 2008), and allowing OVA to translocate into the cytoplasm for cross-presentation (Fig. 3), consistent with its pH-responsive release behavior observed in vitro (Fig. 2F). Effective antigen presentation and subsequent activation of cytotoxic T lymphocyte also require DCs to transition from an immature to a mature state, which was significantly enhanced by Zn^2+^ and Fe^3+^ released from endosomes into the cytoplasm (Fig. 3, Fig. 4). These results align with the previously reported effects of zinc-organometallic framework vaccines and iron oxide nanoparticle-based vaccines in facilitating DC maturation and antigen cross-presentation (Ding et al., 2023; Zhao et al., 2018).

To evaluate the ability of our OVA@LMFZ to elicit robust antigen-specific immunity, we measured antibody levels in mouse plasma. As shown in Fig. 5, exposure to OVA@LMFZ elicited a more potent antigen-specific IgG2a response compared to IgG1, suggesting a potential shift in the Th1/Th2 immune balance toward Th1-dominant immunity. Furthermore, OVA@LMFZ elicited stronger CTL activity in vivo compared to free antigen (Fig. 5E and G). This aligns with previous findings on nanoparticle-based systems for co-delivering antigens and adjuvants, which demonstrated enhanced CD8^+^ T cell immunity than free OVA (Ding et al., 2023). The enhanced proliferation of T cells may also be associated with intracellular zinc released from LMFZ, which activates key signaling molecules involved in immune activation (Schaible and Kaufmann, 2004).

OVA@LMFZ buffered the acidic tumor microenvironment and released metal ions, further enhancing the immune response. Research has revealed that iron oxide nanoparticles influence macrophage function, including apoptosis and pro-inflammatory polarization, thereby contributing to tumor inhibition (Zanganeh et al., 2016). Consistent with these findings, our data demonstrated that OVA@LMFZ effectively induced M1 polarization of macrophages. It has been reported that zinc supplementation raises the number of NK cells in vitro and stimulates greater cytotoxic activity, which potentially associated with the upregulation of GATA-3, a zinc-finger transcription factor crucial role for the development and effector function of both T cells and NK cells (Schaible and Kaufmann, 2004). As shown in Fig. 5H, OVA@LMFZ dramatically elevated the proportion of activated NK cells within the tumor tissue.

To assess the therapeutic efficacy of OVA@LMFZ, we selected melanoma (B16F10-OVA), a highly aggressive and life-threatening malignancy as tumor model. Our findings indicate the potential of OVA@LMFZ as a co-delivery system for OVA and adjuvant to induce a therapeutic immune response against melanoma. Moreover, OVA@LMFZ showed much lower distribution to liver and other tissues, with normal hepatorenal function markers, suggesting a lower risk of systemic toxicity (Fig. S5). While further studies are necessary to comprehensively assess the adjuvant effects of the LMFZ system, these results highlight its potential to effectively enhance immune responses while minimizing the toxicity often associated with adjuvant use.

## 5 Conclusion

In summary, MgZnFe-layered double hydroxide were successfully engineered as a dual-function tumor platform for co-delivering model antigen (OVA) and metal ion adjuvant (Zn^2+^ and Fe^3+^). With its nanoscale dimensions and positive surface charge, the system efficiently targeted lymph nodes and enhanced antigen uptake by dendritic cells. Additionally, its endosomal escape capability ensured effective cross-presentation of the antigen. This co-delivery strategy significantly augmented cytotoxic T lymphocyte activity and inhibited the progression of both primary and distant melanoma (B16F10-OVA) tumors more effectively than free OVA. These results underscore the potential of MgZnFe-layered double hydroxides as a versatile platform for subunit vaccine-based tumor immunotherapy, offering insights into the rational design of nanoparticle-based cancer vaccine systems.

## CRediT authorship contribution statement

**Xue Wang:** Writing – original draft, Methodology, Investigation, Conceptualization. **Lu Liu:** Writing – original draft, Methodology, Investigation. **Yingying Chen:** Writing – review & editing, Visualization, Software, Investigation. **Lirui Jia:** Investigation, Conceptualization. **Yongjun Wang:** Writing – review & editing, Conceptualization. **Wei Jing:** Writing – review & editing, Methodology. **Guangqi Yan:** Writing – review & editing, Validation, Supervision, Project administration, Conceptualization.

## Declaration of competing interest

The authors declare that they have no known competing financial interests or personal relationships that could have appeared to influence the work reported in this paper.

## Data Availability

Data will be made available on request.
